# Metabolomics Reveals the Mechanisms for the Pulmonary Toxicity of *Siegesbeckia orientalis* L. and the Toxicity-Reducing Effect of Processing

**DOI:** 10.3389/fphar.2021.630319

**Published:** 2021-08-09

**Authors:** Ting Jiang, Linsheng Liu, Mi Zhang, Zhiping Qiao, Tingxiu Zhao, Junfang Su, Gang Cao, Tao Su

**Affiliations:** ^1^ International Institute for Translational Chinese Medicine, School of Pharmaceutical Sciences, Guangzhou University of Chinese Medicine, Guangzhou, China; ^2^ Department of Clinical Pharmacology, The First Affiliated Hospital of Soochow University, Suzhou, China; ^3^ School of Basic Medical Sciences, Guangzhou University of Chinese Medicine, Guangzhou, China; ^4^ School of Pharmacy, Zhejiang Chinese Medical University, Hangzhou, China

**Keywords:** *Siegesbeckia orientalis* L., processing with rice wine, pulmonary toxicity, metabolomics, oxidative stress

## Abstract

*Siegesbeckia orientalis* L. (SO) is a commonly used Chinese medicinal herb. It has long been used as a remedy in traditional Chinese medicine (TCM) for symptoms that resemble inflammatory joint disorders. However, it is slightly toxic. According to the TCM theory, processing can reduce the toxicity of the herbs. Here, we performed metabolomics to determine whether processing with rice wine reduces the toxicity of raw SO, and to explore the mechanisms underlying the raw SO–induced toxicity and the toxicity-reducing effect of processing. Our results showed that raw SO has long-term toxicity in rats. It significantly elevated the serum level of LDH and caused histopathological damages in the lung tissues. It is worth noting that the LDH level in the PSO group was lower than that in the raw SO group, and the damages in lung tissues were relatively mild in PSO-treated rats, suggesting that processing reduces the pulmonary toxicity of the raw. Moreover, a total of 32 significantly changed metabolites were identified. Based on the MetaboAnalyst pathway analysis, we found that two characteristic metabolic pathways including alanine, aspartate and glutamate metabolism and glycerophospholipid metabolism were only changed in the raw SO group, while histidine metabolism was only changed in the PSO group, which suggests that induction of oxidative stress contributes to raw SO–induced pulmonary toxicity, and free radical scavenging might be responsible for the toxicity-reducing effect of processing. Our data shed new light on how raw SO induces pulmonary toxicity and how the toxicity can be reduced by processing. This study not only provides scientific justifications for the traditional processing theory of SO, but also helps to optimize the processing protocol and the clinical drug combination of SO.

## Introduction

*Siegesbeckia orientalis* L. (SO), a traditional Chinese medicinal herb, was recorded to be able to eliminate the wind-dampness and soothe painful joints (the State Commission of Chinese Pharmacopoeia, 2020), and to be slightly toxic in *Xinxiu Bencao* (657–659 A.D., Tang Dynasty in China). SO is commonly used in managing traditional Chinese medicine (TCM) symptoms that resemble joint inflammatory disorders such as rheumatoid arthritis (RA). Chemical studies have revealed that SO contains terpenoids, glycosides, *etc*. Toxicological studies have demonstrated that the water extract of SO has acute toxicity in animals (Guan et al., 2007). In addition, reversible pulmonary toxicity of SO is observed in a subacute toxicity study in mice (Guan et al., 2008). SO is used as a long-term drug for treating chronic diseases such as RA and hypertension in clinical practice. However, up to now, the long-term toxicity of SO remains unknown, and the mechanisms underlying the raw SO–induced toxicity is still not fully understood.

Raw Chinese medicinal herbs are subjected to processing before they are used for clinical prescriptions or for preparing proprietary Chinese medicines. According to the TCM theory, processing can reduce the toxicity of the herbs. As recorded in Chinese Pharmacopoeia, SO is usually processed with rice wine. In our previous study, we have demonstrated that PSO is less toxic than raw SO as demonstrated in our tested human embryonic lung cells (MRC-9) (Su et al., 2014). However, up to now, the underlying toxicity-reducing mechanisms of processing are not known.

Chinese medical herbs have multiple chemical components and the multi-target nature (Yu et al., 2012), hence, using conventional research approaches such as biochemical and histological analyses to elucidate the mechanisms for herbal toxicities and the toxicity-reducing effect of processing have limitations. Metabolomics is a systematic approach for analyzing the small-molecule metabolites using various analytical methods, it can figure out the biological implications of the metabolites using bioinformatics means, and it has emerged as a powerful approach for this kind of studies (Tan et al., 2014). Here, for the first time, we explored the mechanisms underlying the raw SO–induced pulmonary toxicities and the toxicity-reducing effect of processing using metabolomics.

## Materials and Methods

### Chemicals and Reagents

Raw SO (the dried aerial part of *Siegesbeckia orientalis* L.) was collected from Ganzhou (Jiangxi province, China). Rice wine was purchased from Zhejiang Tapai Shaoxing Wine Co., Ltd. (Zhejiang province, China). LC–MS grade acetonitrile, methanol, and formic acid (FA) were purchased from CNW Technologies (Germany). Kirenol was purchased from Shanghai Yuanye Bio-Technology Co., Ltd. (Shanghai, China). Adonitol was purchased from Sigma-Aldrich (MO, United States). Milli-Q water was prepared using a Milli-Q system (Millipore, MA, United States). The commercial kits including creatine kinase (CK), lactate dehydrogenase (LDH), urea, aspartate aminotransferase (AST), and alanine aminotransferase (ALT) were purchased from Shenzhen Mindray Bio-Medical Electronics Co., Ltd. (Guangdong province, China). Malondialdehyde (MDA) was purchased from Nanjing Jiancheng Bioengineering Institute (Jiangsu province, China).

### Preparation of Raw SO and PSO Extracts

The authentication of SO was confirmed in accordance with the corresponding monograph in CP 2020 (2020 edition of CP) by the corresponding author. Voucher specimens of raw SO (No. 20180510) and rice wine were deposited at the International Institute for Translational Chinese Medicine, Guangzhou University of Chinese Medicine.

PSO preparation: As shown in our previous study, 100 ml of rice wine (containing 15% ethanol): water/20:80 (v/v) was sprayed onto 100 g SO and allowed to be completely absorbed in a covered container for 1.5 h. Subsequently, the mixture was steamed for 5 h, cooled, and then dried at 40°C in a Memmert UFE500 oven (Su et al., 2014).

Raw SO and PSO extracts preparation: Raw SO or PSO (500 g) was reflux-extracted twice with water (1:10, w/v) for 2 h each. The combined extracts were filtered after cooling and then concentrated under reduced pressure to remove the water. The powdered SO (yield: 18.5%) and PSO (yield: 14.9%) extracts were obtained by lyophilizing of the concentrated samples with a VirTis Freeze Dryer (The VirTis Company, New York, United States). Powdered SO or PSO was dissolved in water to prepare raw SO and PSO solution. Detailed information of the high performance liquid chromatography (HPLC) analysis was shown in Supplementary Materials. Results showed that the contents of kirenol in raw SO and PSO were 0.13 and 0.07%, respectively (Supplementary Figure S1).

### Animals and Treatments

A total of 24 male SD rats (200 ± 20 g, 6–8 weeks) were obtained from the Laboratory Animal Center of Southern Medical University [License number: SCXK (GZ)2016-0041; Guangzhou, China] and kept in the animal laboratory [License number: SYXK (GZ) 2019-0144] at the International Institute for Translational Chinese Medicine, Guangzhou University of Chinese Medicine (Guangzhou, China). Animal experiments were approved by the Guangzhou University of Chinese Medicine Animal Care and Use Committee (Guangzhou, China) and conducted according to the ethical standards and national guidelines. Every effort was made to reduce the number of animals used and minimize their pain and distress. After one week of acclimatization, rats were randomly divided into three groups (*n* = 8), and daily intragastrically administered an equal volume of the vehicle, raw SO or PSO extract at a dosage of 5 g/kg/day for 6 months. The experimental design was shown in Supplementary Figure S2.

### Animal Sample Preparation

Blood samples were collected from the retro-orbital venous plexus each month. The blood samples were centrifuged at 3500 rpm for 10 min after standing for 2 h at 4°C. The serum was then transferred into new tubes and stored at −80°C for further analysis. A portion of the collected serum was used for routine biochemical analyses of the creatine kinase (CK), lactate dehydrogenase (LDH), urea, aspartate transaminase (AST), alanine transaminase (ALT), and plasma malondialdehyde (MDA) levels according to the manufacturer’s instructions of the respective commercial assay kits. At the end of the experiment, all rats were sacrificed followed by blood collection. Fresh cardiac, hepatic, lung, and renal tissues were dissected and fixed in 10% neutral buffered formaldehyde at 4°C for paraffin embedment. Organ samples (4 μm) were sectioned and stained with H&E, and finally examined under light microscopy.

100 μL of serum was transferred and 400 μL extract solution (acetonitrile: methanol = 1:1) containing internal standard (adonitol, 1 μg/ml) was added. After 30 s vortex, samples were sonicated for 5 min in an ice-water bath. Then the samples were incubated at −40°C for 1 h and centrifuged at 10,000 rpm for 15 min at 4°C. 400 μL of supernatant was transferred to a fresh tube and dried in a vacuum concentrator at 37°C. Then, the dried samples were reconstituted in 100 μL of 50% acetonitrile by sonication on ice for 10 min. The constitution was then centrifuged at 13000 rpm for 15 min at 4°C, and 60 μL of supernatant was transferred to a fresh glass vial for LC/MS analysis. The quality control (QC) sample was prepared by mixing an equal aliquot of the supernatants from all the samples.

### LC-TOF-MS/MS Analyses

The UHPLC separation was carried out using an ExionLC Infinity series UHPLC System (AB Sciex), equipped with a UPLC BEH Amide column (2.1 × 100 mm, 1.7 μm, Waters). The mobile phase consisted of 25 mmol/L ammonium acetate and 25 mmol/L ammonia hydroxide in water A) and acetonitrile B). The analysis was carried with an elution gradient as follows: 0–0.5 min, 95%B; 0.5–7.0 min, 95%–65% B; 7.0–8.0 min, 65%–40% B; 8.0–9.0 min, 40% B; 9.0–9.1 min, 40–95% B; 9.1–12.0 min, 95% B. The column temperature was 25°C. The auto-sampler temperature was 4°C, and the injection volume was 2 μL (pos) or 3 μL (neg), respectively.

The TripleTOF 5600 mass spectrometry (AB Sciex) was used for its ability to acquire MS/MS spectra on an information-dependent basis (IDA) during an LC/MS experiment. In this mode, the acquisition software (Analyst TF 1.7, AB Sciex) continuously evaluates the full scan survey MS data as it collects and triggers the acquisition of MS/MS spectra depending on preselected criteria. In each cycle, the most intensive 12 precursor ions with intensity above 100 were chosen for MS/MS at collision energy (CE) of 30 eV. The cycle time was 0.56 s. ESI source conditions were set as following: gas 1 as 60 psi, gas 2 as 60 psi, curtain gas as 35 psi, source temperature as 600°C, declustering potential as 60 V, and ion spray voltage floating (ISVF) as 5,000 V or −4,000 V in positive or negative modes, respectively.

### Sequence Analysis

The pooled quality control (QC) sample was analyzed at the beginning, the end, and randomly through the analytical run to monitor the stability of sequence analysis. The typical batch sequence of serum samples consisting of consecutive analysis cycles of 1 QC serum sample (at the beginning of the study) follow by 8 unknown serum samples. Meanwhile, samples were analyzed in a random order for a normal good practice. An identical sequence was repeated to complete the total set of injections (*n* = 28, including QCs) analyzed in less than one day per mode.

### LC–MS Data Processing

Rat serum samples were analyzed by an untargeted metabolomics approach based on UPLC-Q-TOF/MS. The MS raw data (.wiff) files were converted to the mzXML format by ProteoWizard, and processed by R package XCMS (version 3.2). Principle component analysis (PCA) and orthogonal projection to latent structures discriminant analysis (OPLS-DA) were used to assess the data. Major metabolites were screened and identified using in-house libraries and the METLIN (http://metlin.scripps.edu) database, the MassBank database (http://www.massbank.jp/), the Human Metabolome Database (HMDB) (http://www.hmdb.ca/), or the LipidMaps database (http://www.lipidmaps.org) based on accurate mass measurements (mass errors <5 ppm) and MS/MS fragment information.

### Statistical Analysis

Results of the biochemical assays were presented as Mean ± SEM. These data were analyzed by one-way ANOVA followed by the Dunnett’s multiple comparisons using GraphPad Prism version 7.0 (GraphPad Software, San Diego, CA, United States). *p* < 0.05 was considered statistically significant.

## Results

### Biochemical Changes

**FIGURE 1:**
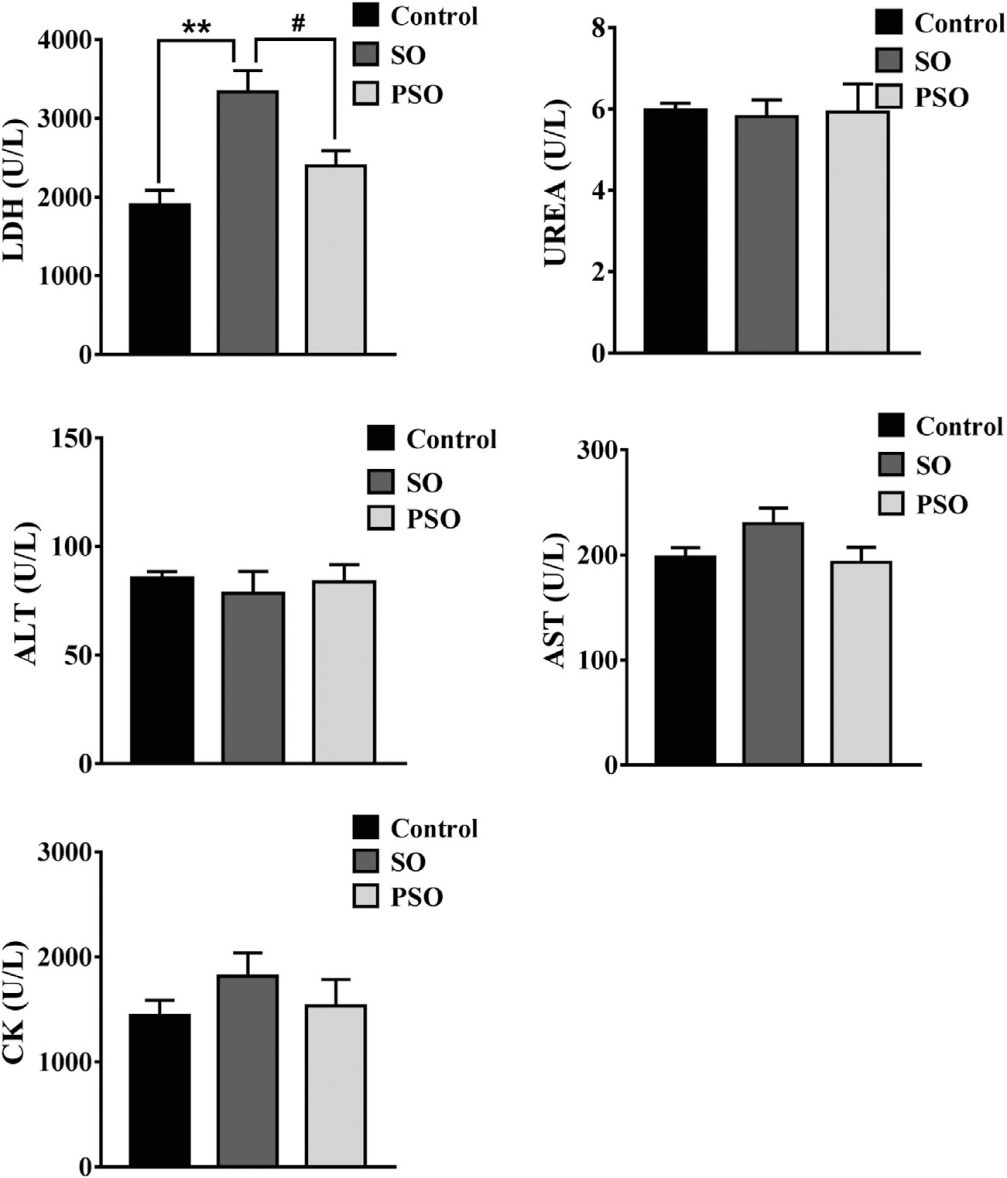
Biochemical parameters in the serum of vehicle-, raw SO- and PSO-treated rats at month 6. **p < 0.01 *vs*. control; ^#^ *p* < 0.05 *vs.* raw SO.

The serum levels of AST, ALT, Urea, CK, and LDH were measured in each rat every month. ALT and AST are the markers of hepatic damage; Urea is a marker of renal damage; CK is a marker of cardiac damage. However, currently, no specific markers were used for evaluating the lung damage. It is known when an organ is damaged, LDH will be released into the circulation, which is known as a biomarker for tissue damage. Moreover, it has also been reported that the measurement of LDH activity in lung lavage fluid can assess the extent of cell damage (Gao et al., 2013). Here, we found that the serum level of LDH in raw SO group was significantly higher than that in the control group after 6-months treatment (*p* < 0.01). It is worth noting that, the LDH level in the PSO group was lower than that in the raw SO group (*p* < 0.05) (Figure 1), suggesting that processing significantly reduced the LDH level in the serum. Although raw SO increased the levels of CK and AST in the serum when compared to the control groups, they did not reach statistical significance. In addition, we also found that both raw SO and PSO did not significantly affect the serum levels of ALT, AST, Urea, and CK in the earlier stage of the treatment, suggesting that the treatments at the early stage have no obvious liver, kidney and heart toxicities were observed in these rats (Supplementary Figure S3).

### Histopathological Changes

**FIGURE 2:**
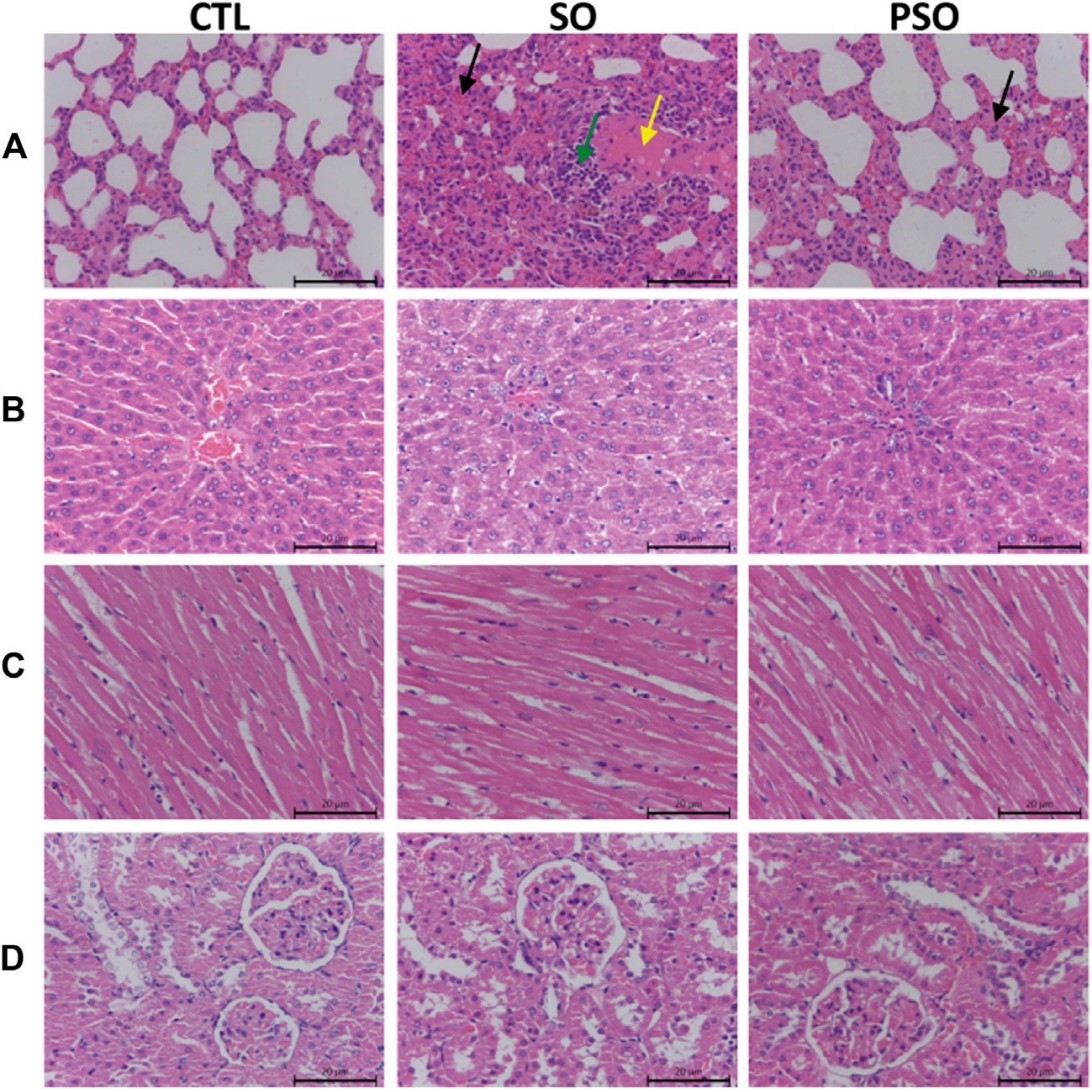
Histopathological examinations of the lung, liver, heart, and kidney tissues in vehicle-, raw SO- and PSO-treated rats. H&E staining, 200×. **(A)** Lung tissue of each group: yellow arrow represents serous exudation in the alveolar cavities, green arrow represents inflammatory cell infiltration, black arrow represents the thickened alveolar septum, and the capillaries are dilated and congested; **(B)** liver tissue of each group; **(C)** heart tissue of each group; and **(D)** kidney tissue of each group.

H&E staining was used to further examine the treatment-induced toxicities in heart, liver, lung, and kidney tissues. We found that histopathological damages were observed in the lung tissues of the raw SO-treated rats as indicated by a lot of inflammatory cells infiltration, and along with the cells edema; while, the damages were relatively mild in PSO-treated rats. Liver and heart sections showed little pathological damages in the raw SO group (Figure 2), suggesting that long-term administration of raw SO may not cause obvious damages in the liver and heart when compared to the lung. Combining the pathological examination and the biochemical results, we strongly suggest that raw SO mainly causes pulmonary toxicity, which can be significantly reduced by processing.

### Identification of Differential Metabolites

**FIGURE 3:**
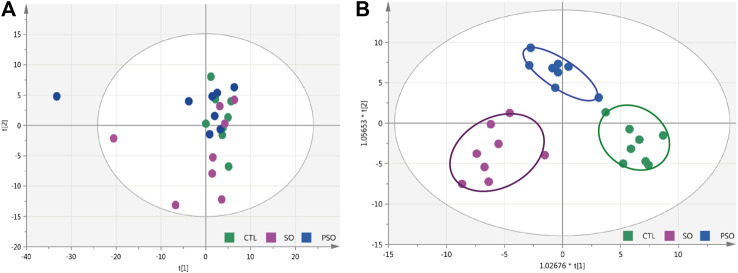
PCA and OPLS-DA modeling were used to differentiate the rat serum metabolomic patterns after raw SO and PSO treatment. **(A)** PCA modeling; **(B)** OPLS-DA modeling. Control group (green), raw SO group (violet), and PSO group (blue).

Total ion chromatogram (TIC) chromatograms of the serum samples were obtained from vehicle-, raw SO-, and PSO-treated rats (Supplementary Figure S4). Metabolomics chromatograms obtained through LC-Q-TOF/MS using the ESI^+^ and ESI^−^ modes revealed distinct differences. In total, 32 altered metabolites were authentically identified, including amino acids, lipids, hydrocarbons, and nucleotides. The heatmap figure showed the detailed information (Supplementary Figure S5). Based on the information obtained from LC-Q-TOF/MS, general OPLS-DA and PCA models were calculated to detect the significantly changed metabolites (VIP>1 or *p* < 0.05) among the control, raw SO and PSO groups. PCA scores (PC1: 14.4%, PC2: 12.4%, PC3: 9.32%) showed that the three groups were clustered (Figure 3A). The multiple pattern recognition method of partial least squares discriminant analysis was adopted based on the basis of the metabolic changes in the three groups as revealed by TIC chromatograms. As shown in the PLS-DA scores plot (Figure 3B, R_2_X = 0.41, R_2_Y = 0.841, Q_2_ = 0.388), we found that the distribution of each group was concentrated, and the distance between the raw SO group and control group was far, while that between the PSO group and the control group was comparatively shorter, suggesting that significant changes of the metabolites in the serum levels are shown after the administration of raw SO in rats.

**FIGURE 4:**
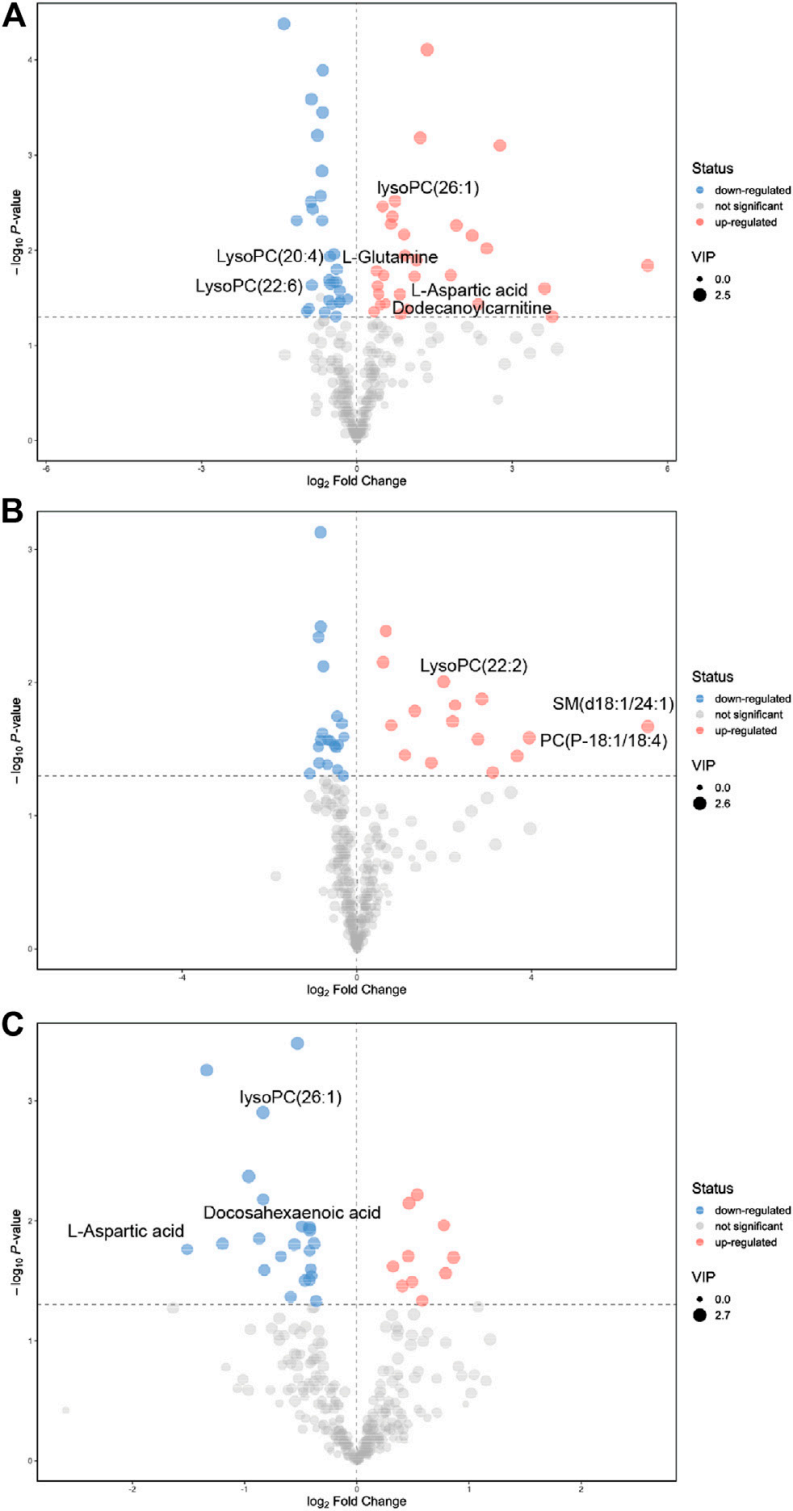
Significantly changed metabolites were shown in the volcano figure. **(A)** raw SO *vs.* control, **(B)** PSO *vs.* control, and **(C)** PSO *vs.* raw SO.

As shown in Table 1, a total of 32 metabolites were significantly changed when compared to the control group, among them, 10 metabolites were significantly downregulated including LysoPC(20:4), LysoPC(22:6), and glucosylsphingosine, while 22 metabolites were significantly upregulated including L-glutamine, L-aspartic acid, and phosphorylcholine. For the PSO group, we found that 5 metabolites were significantly downregulated including acetylcysteine, D-tryptophan, and gamma-linolenic acid, while 14 metabolites were significantly upregulated including PC (16:0/16:0), PE(16:0/20:1), and PE (22:5/22:6). Interestingly, 14 metabolites which obviously altered in the raw SO group, showed no significant differences in the PSO group, which is similar to the control group. The volcano figure can also show the metabolites fluctuation of various compounds (Figure 4). These altered metabolites may partly explain the toxicity that is caused by raw SO and the toxicity-reducing effect of processing.

**TABLE 1:** Significantly altered metabolites in the serum samples of vehicle-, raw SO- and PSO-treated rats. ↑: upregulation; ↓: downregulation; —: no significant change.

Super-pathway	Sub-pathway	No.	Metabolites	SO vs. CTL	PSO vs. CTL
Amino acid	Cysteine metabolism	1	Acetylcysteine	↓	↓
	Tryptophan	2	D-Tryptophan	↓	↓
	Metabolism	3	L-Glutamine	↑	—
	Arginine biosynthesis	4	L-Aspartic	↑	—
Lipid	Glycerophospholipid metabolism	5	Phosphorylcholine	↑	—
		6	PC (16:0/16:0)	↑	↑
		7	PE (16:0/20:1)	↑	↑
		8	PE (22:5/22:6)	↑	↑
		9	PE (22:6/P-18:0)	↑	↑
		10	PE-NMe (16:0/16:0)	↑	↑
		11	PC (16:0/16:0)	↑	↑
		12	PC (P-18:1/18:4)	↑	↑
	Lysophospholipid metabolism	13	LysoPA (18:1)	↑	—
		14	LysoPC (20:4)	↓	—
		15	LysoPC (22:2)	↑	↑
		16	LysoPC (22:6)	↓	—
		17	LysoPC (24:1)	↑	↑
		18	lysoPC (26:1)	↑	—
		19	LysoPE (18:1)	↑	—
	Diglyceride metabolism	20	DG (20:4/18:2/0:0)	↓	↓
	Glycosphingolipids	21	Glucosylsphingosine	↓	—
	Sphingomyelin metabolism	22	SM (d18:1/16:0)	↑	↑
		23	SM (d18:1/24:1)	↑	↑
	Fatty acid metabolism	24	Docosahexaenoic acid	↑	—
		25	Hexadecanedioic acid	↑	—
		26	Hexadecanoic acid	↑	↓
		27	Oleamide	↓	↑
		28	Gamma-Linolenic acid	↓	↓
		29	Myristic acid	↓	—
Hydrocarbon	Oxidation of fatty acids	30	Vaccenyl carnitine	↑	↑
		31	Dodecanoylcarnitine	↑	—
Nucleotide	Purine metabolism	32	Guanine	↓	—

### Metabolic Pathway and Network Function Analyses

**FIGURE 5:**
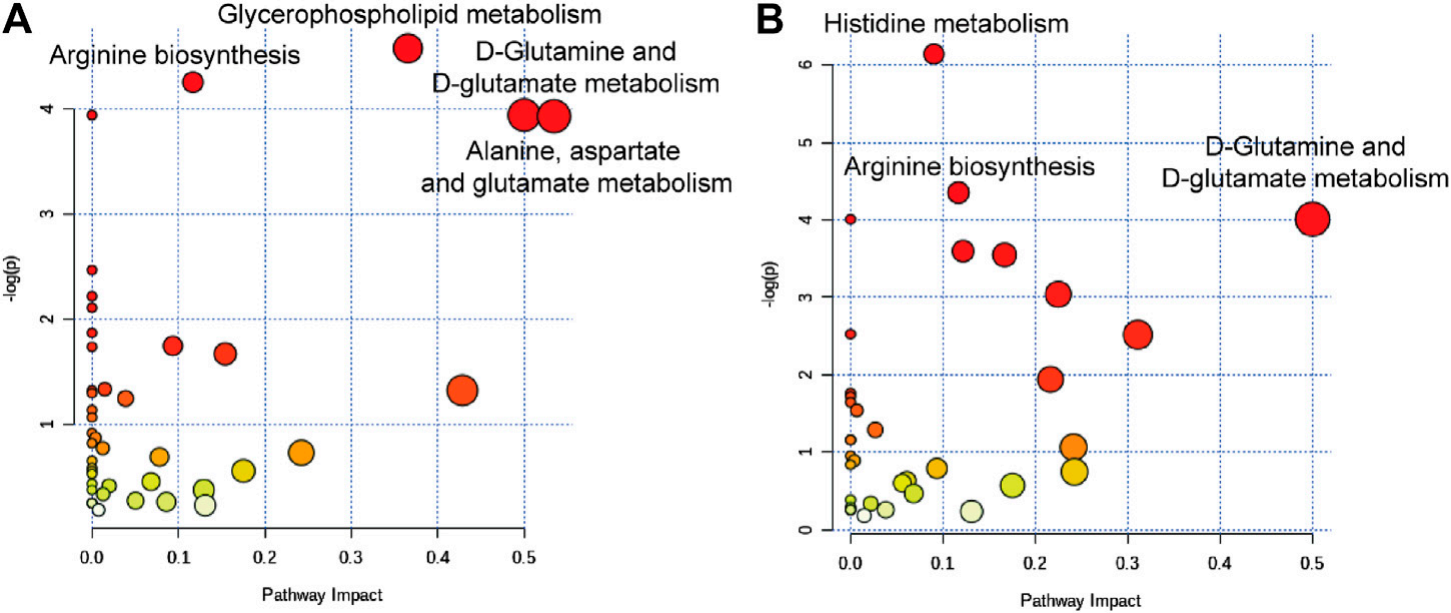
Summary of the metabolic pathways analyzed with MetaboAnalyst software. **(A)** The most significantly altered pathways in the raw SO group, raw SO *vs.* control. **(B)** The most significantly altered pathways in the PSO group, PSO *vs.* control.

To further understand the correlation between the candidate metabolites and the biological association networks, we performed pathway analysis using MetaboAnalyst (https:// www.metaboanalyst.ca/) pathway analysis. As shown in Figure 5, four significant metabolic pathways including arginine biosynthesis, glycerophospholipid metabolism, D-glutamine, and D-glutamate metabolism, and alanine, aspartate, and glutamate metabolism were identified in the serum samples of raw SO-treated rats; while, histidine metabolism, arginine biosynthesis, and D-glutamine and D-glutamate metabolism were identified in the serum samples of PSO-treated rats.

Among them, two characteristic metabolic pathways alanine, aspartate and glutamate metabolism and glycerophospholipid metabolism were only changed in the raw SO group. It was reported that both glycerophospholipid metabolism (Wang et al., 2019) and arginine biosynthesis (Mizrahi and Warner, 2018) are closely related to oxidative stress. Therefore, we speculated that the raw SO–induced oxidative stress response may underlie the pulmonary toxicity. In addition, histidine metabolism was only changed in the PSO group. Studies suggested that histidine, an amino acid that is essential to humans, exerts favorable cytoprotective effects against oxidative stress as have been demonstrated both *in vivo* and *in vitro*, which has been shown to scavenging free radicals (Vera-Aviles et al., 2018). Hence, we speculated that free radical scavenging may be responsible for the toxicity-reducing effect of processing.

### Verification of the Mechanisms Underlying the Raw SO-Induced Pulmonary Toxicity and the Toxicity-Reducing Effect of Processing

**FIGURE 6:**
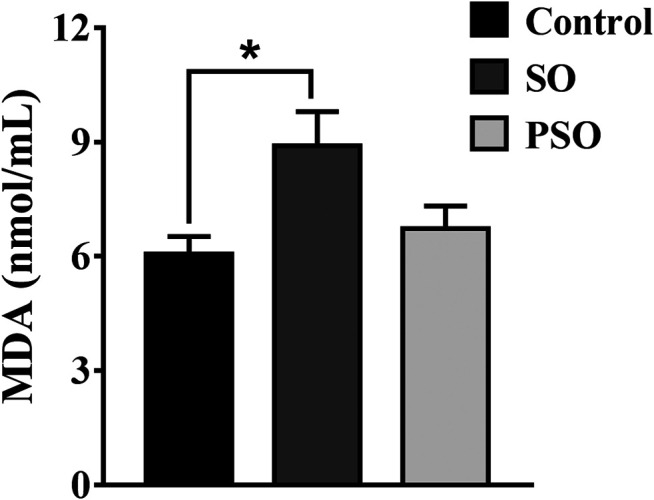
MDA contents in the serum of vehicle-, raw SO- and PSO-treated rats. *p < 0.05 vs. control.

In an attempt to verify the highlighted biological functions, we examined the MDA contents in the serum samples of vehicle-, raw SO-, and PSO-treated rats. MDA, a classic biomarker of oxidative stress, is produced by free radicals in the body (Lykkesfeldt, 2007). It is also widely used as an indicator of the free radical level. As shown in Figure 6, we found that raw SO treatment significantly increased the serum MDA content when compared to the vehicle control group (*p* < 0.05), suggesting that raw SO causes oxidative stress in the rats. While the MDA content in the PSO group was lower than that in the raw SO group (*p* < 0.05), and no significant difference was observed between the control and PSO groups, suggesting that processing reduces the oxidative stress-causing effects of raw SO or processing enabled the herb to possess a free radical scavenging property. These findings also suggest that free radical scavenging may be responsible for the pulmonary toxicity-reducing effect of processing.

## Discussion

Raw SO is toxic, studies have demonstrated that the water extract of SO has acute and subacute toxicities (Guan et al., 2007; Guan et al., 2008). However, the long-term toxicity of SO is unknown. It is worth noting that in clinical practice SO is commonly used for treating chronic diseases, such as RA and hypertension, which require long-term treatment. Therefore, it is necessary to explore the long-term toxicity of this herb. In this study, we explored the long-term toxicity of SO, and revealed the molecular mechanisms underlying the raw SO–induced toxicity. In the future, toxicity studies in the disease models will be conducted.

According to the TCM theory, processing can reduce the toxicity of the herbs. The theory has been supported by modern toxicology studies. In our previous study, PSO was demonstrated less toxic than raw SO in our tested MRC-9 cells (Su et al., 2014). However, the mechanisms of raw SO–induced toxicities and the toxicity-reducing effect of processing are still not fully understood. Here, we conducted the metabonomics study to explore the mechanisms that underlie the raw SO–induced pulmonary toxicity and the toxicity-reducing effect of processing.

In this study, a total of 32 metabolites are significantly altered including amino acids, lipids, hydrocarbons and nucleotides, they were authentically identified in the serum samples of vehicle-, raw SO-, and PSO-treated rats (Table 1). In the raw SO group, 22 metabolites were upregulated, while 10 metabolites were downregulated. In the PSO group, 13 metabolites were upregulated, 5 metabolites were downregulated, and the other 14 metabolites are not significantly different when compared to the healthy rats.

Among all the metabolites, 8 characteristic metabolites including L-glutamine, L-aspartic acid, phosphorylcholine, lysoPA (18:1), lysoPC (26:1), lysoPE (18:1), docosahexaenoic acid, and dodecanoylcarnitine were only upregulated in the raw SO group, and they do not show changes in the PSO group. Among them, two were amino acids. The observed changes in amino acid contents should be due to the modulation of the endogenous amino acid metabolism system, but not the loading of the exogenous amino acid. In our experiments, we had fasted the rats for 12 h before blood sample collection. The half-lives of exogenous amino acids in rats are very short (Su et al., 2016). Hence, the exogenous amino acids should have been eliminated within 12 h and cannot be detected in the serum. Glutamine is metabolized by glutaminase-1 (GLS1) to form the gas NH_3_. NH_3_ stimulated the production of mitochondrial reactive oxygen species (ROS) which causes the activation and translocation of the NF-E2-related factor-2 transcription factor (Nrf2) into the nucleus, where it binds to the antioxidant responsive element (ARE) in the promoter region of the gene to trigger HO-1 transcription (Durante, 2019). It is reported that glutamine metabolism is required for collagen protein synthesis in lung fibroblasts (Hamanaka et al., 2019), and excessive proliferation of lung fibroblasts could cause lung toxicity (Huang et al., 2012). Here, in this study, we found that glutamine was only upregulated in the raw SO group, suggesting that raw SO was more toxic than PSO. Aspartic acid is an α-amino acid. The study reports that aspartic acid at high dose levels has toxicity in kidneys and salivary glands in Fischer rats (Tada et al., 2008). Docosahexaenoic acid (DHA) is a polyunsaturated ω-3 fatty acid. Some studies suggest that the elevated levels of DHA are associated with decreased risk of dementia, while, other studies reported that DHA micelles stabilized protofibrillar Aβ42 to prevent further fibrillization (Hashimoto et al., 2008). Unfortunately, these protofibrils induced toxicity in PC-12 cells, suggesting that DHA may act as a double-edged sword. Depending on the context, DHA may actually exacerbate the pathology of Alzheimer’s disease (Cole and Frautschy, 2006). Thus, too high or too low concentration of DHA in raw SO-treated rats may cause physiological changes. In addition, by comparing the concentration of dodecanoylcarnitine in the tissues and the urine of healthy and cancer patients, the dodecanoylcarnitine concentrations are higher in both cancer tissues (compared with the paired normal tissue) and in urine of cancer patients (compared with control urine), suggesting that dodecanoylcarnitine is highly expressed in an abnormal physiological state (Niziol et al., 2018), which also implies that raw SO treatment changes the physiological state and finally upregulated the content of dodecanoylcarnitine in the rats. Phosphorylcholine (PC), the polar headgroup of the membrane phospholipid phosphatidylcholine, which is a pro-inflammatory epitope in OxPLs and is recognized as a danger-associated molecular pattern by the innate immune system (Miller et al., 2011). Thus, elevation of this pro-inflammatory molecule in raw SO indicates that it has a strong pro-inflammatory effect, which may partly explain the toxicity that is caused by raw SO, and the reason for our observed inflammatory-cell infiltration in the lung tissues of raw SO-treated rats.

Besides the above eight metabolites that were only upregulated in the raw SO-treated group, another four metabolites were only downregulated in the raw SO group but not in the PSO group. Take glucosylsphingosine as an example, glucosylsphingosine is a key biomarker of Gaucher disease (Murugesan et al., 2016). It is believed to be responsible for macrophagic organ infiltration and the subsequent development of organomegaly, which could cause hematological and visceral changes in animals (Lukas et al., 2017). Myristic acid, is also known as tetradecanoic acid, which is a side chain of phorbol myristate acetate (PMA). It activates human polymorphonuclear leukocytes to produce oxygen radicals more potently than PMA does (Tada et al., 2009). Although the levels of these two metabolites were downregulated in the raw SO group, we know that the organism is a balanced environment, and either too high or too low will have some effects. Whether these changes underlie the toxicity caused by raw SO deserves further investigation.

In addition, 12 metabolites including PE (16:0/20:1), PE (22:5/22:6), and PC (P-18:1/18:4) were upregulated; while, 4 metabolites including acetylcysteine, tryptophan and DG (20:4/18:2/0:0) were downregulated in both raw SO and PSO groups, which may explain the common efficacies of both raw SO and PSO.

## Conclusion

In summary, we for the first time demonstrated that processing with rice wine significantly reduced the long-term toxicity of raw SO, which supports the TCM theory that “processing can reduce the toxicity of raw SO.” Induction of oxidative stress underlies the raw SO–induced pulmonary toxicity, and free radical scavenging was, at least in part, responsible for the toxicity-reducing effect of processing. Our study sheds new light on the mechanisms underlying the raw SO–induced pulmonary toxicity and the toxicity-reducing effect of processing with rice wine. This study not only provides a scientific justification for the traditional processing theory but also should guide rational and safe clinical applications of SO by helping in optimizing its processing procedure and clinical compatibility.

## Data Availability

The original contributions presented in the study are included in the article/Supplementary Material; further inquiries can be directed to the corresponding authors.
