# Supplementary figures and images for: Metabolomics Reveals the Mechanisms for the Pulmonary Toxicity of Siegesbeckia orientalis L. and the Toxicity-Reducing Effect of Processing
Source: Front Pharmacol. 2021 Aug 9;12:630319. doi: 10.3389/fphar.2021.630319 (PMC8381750; doi:10.3389/fphar.2021.630319)

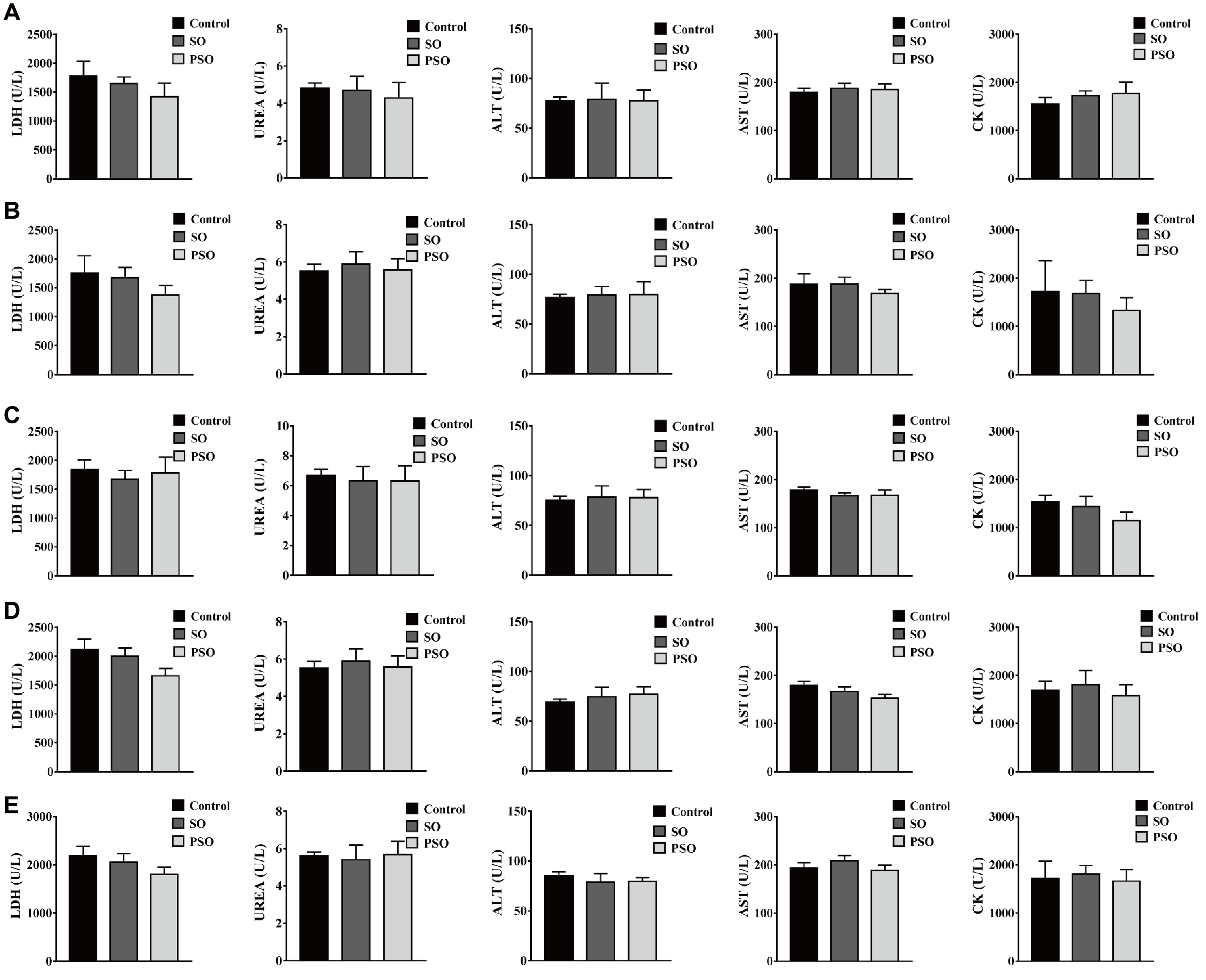

Supplement: Supplementary file 1 [file Image3.TIF]

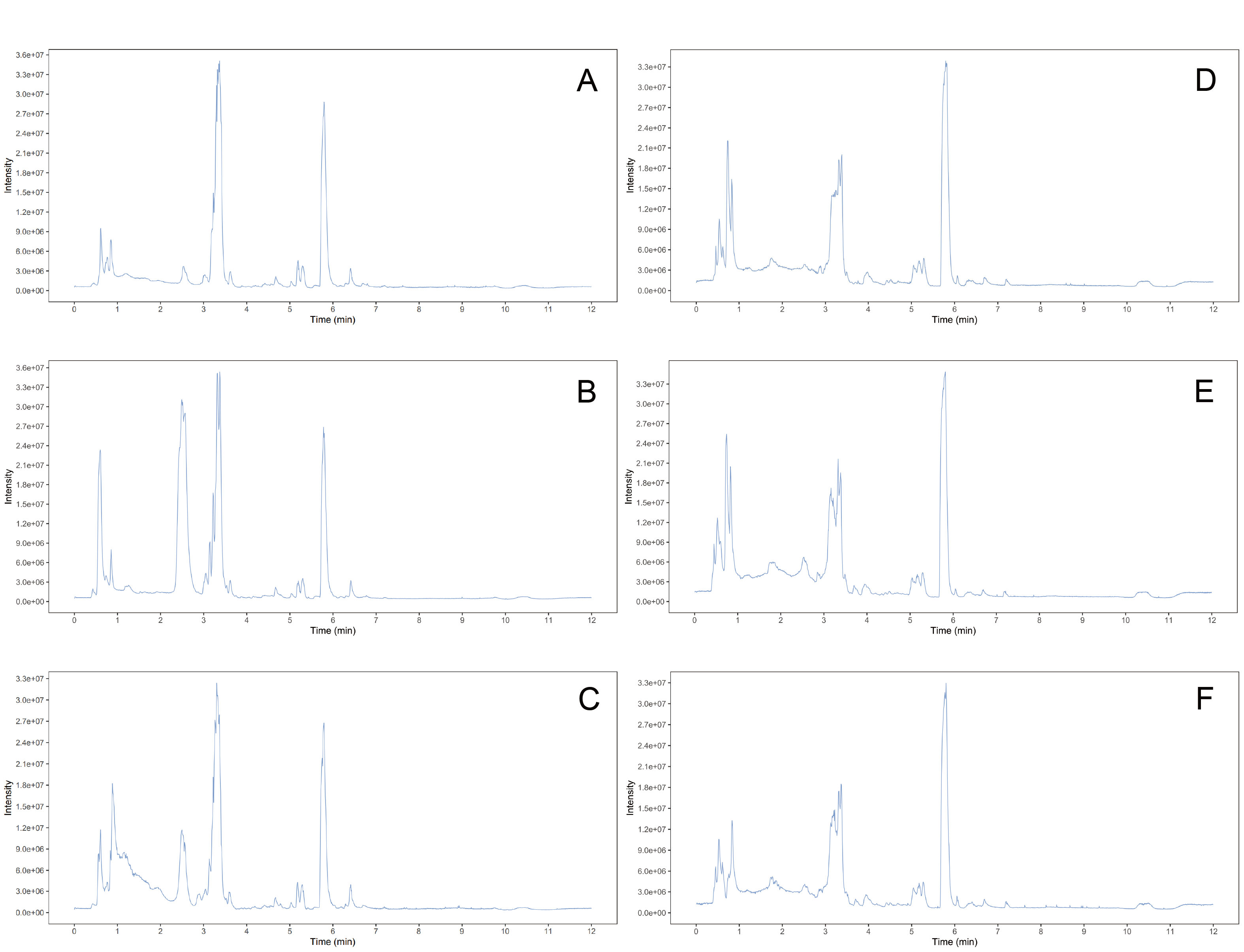

Supplement: Supplementary file 2 [file Image4.TIF]

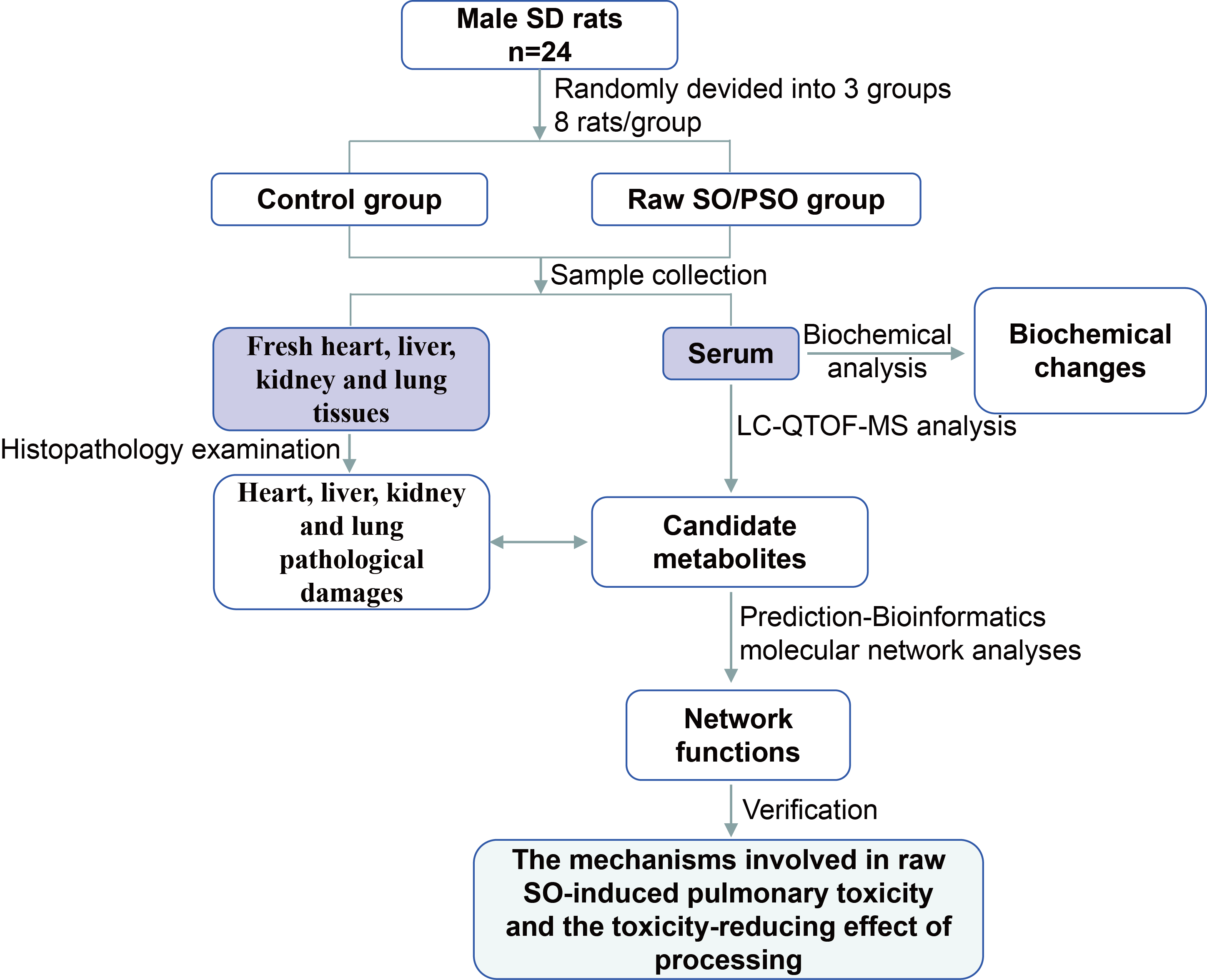

Supplement: Supplementary file 3 [file Image2.TIF]

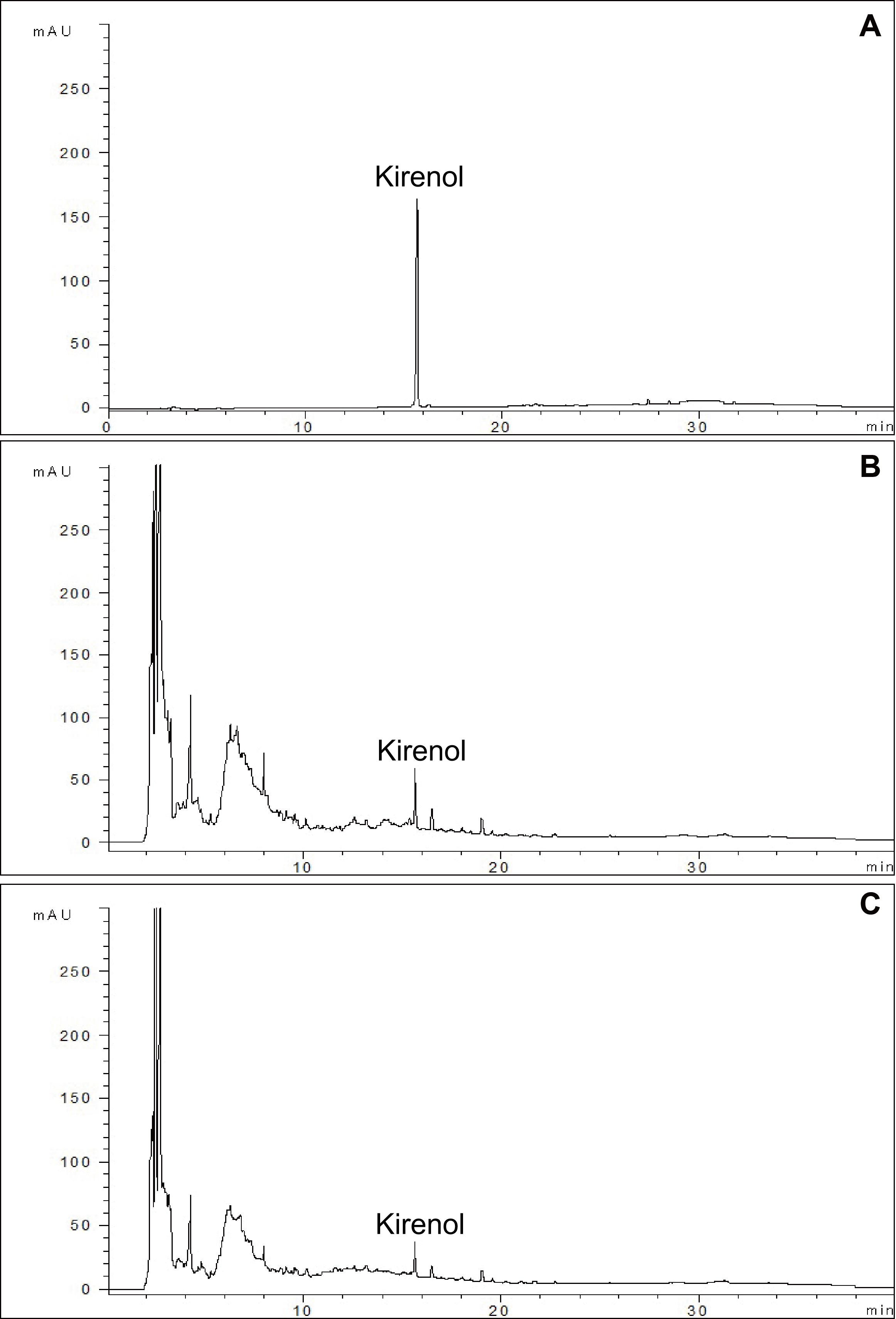

Supplement: Supplementary file 4 [file Image1.TIF]

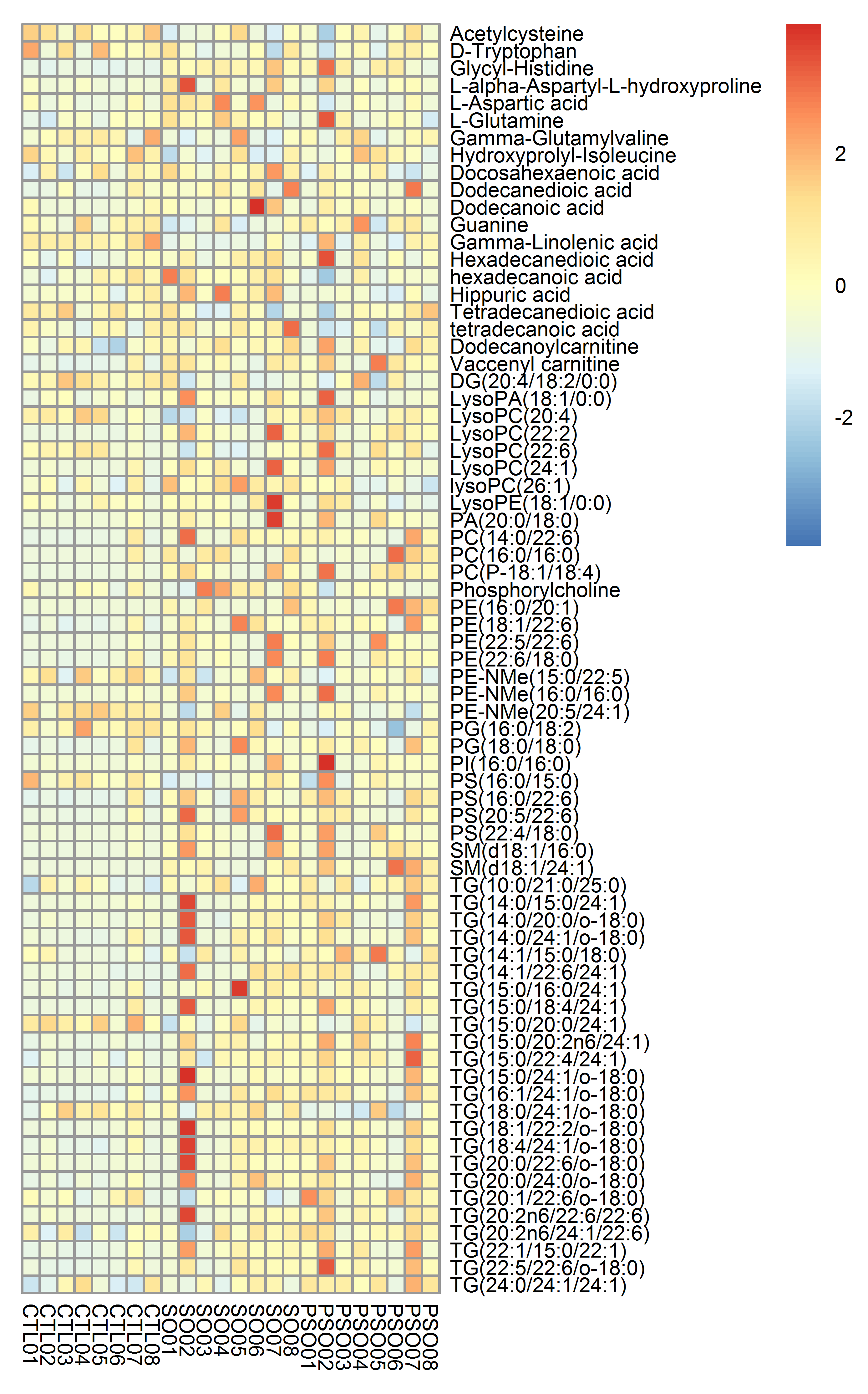

Supplement: Supplementary file 5 [file Image5.TIF]
